# Psychopathology in Art—Meditation on Francis Bacon

**Published:** 1989-08

**Authors:** Michael Wilson

**Affiliations:** Hon. Consultant Surgeon, Southmead Hospital, Bristol


### Psycho-Pathology in Art—Meditation on Francis Bacon

**Michael Wilson, ChM, FRCS**
Hon. Consultant Surgeon, Southmead Hospital, Bristol

Like most of us, for years I have contemplated with revulsion and fascination, reproductions of the pictures of Francis Bacon. I had never actually seen an original until I went with the Friends of Bristol Art Gallery to Sutton Place, that magnificent Tudor Mansion, formerly the home of the American millionaire oil magnate and art collector, J. Paul Getty. It is now leased by another American art collector, Mr Stanley J. Seeger and houses his collection of pictures by contemporary artists. Occupying pride of place in the Great Hall is a Triptych by Francis Bacon. From a distance it looks well, with its warm orange colouring against the background of oak panelling and the pleasing symmetry of its forms. The unwary however, as he approaches it is in for a shock. In the central picture is a table on which two apparently male figures, prone, distorted and partially fused seem to be locked in embrace. Reclining on tables in the two outer pictures are also male figures. On one side, turned towards the viewer, he is deformed with smeared features and apparently armless, on the other side, the figure turning away from him displays a long red gash on his back. The impact of these repulsive images is enhanced by the careful technique with which they are painted. Initial reactions are ribald mingled with disgust. One stands in front of a picture such as Bacon's Triptych and ask oneself—why? Why does Bacon paint like this?—and even more incomprehensible—why do the gurus of the Art Establishment accord him their homage?

I heard Paul Feiler, at the time Head of painting at the West of England School of Art say of abstract painting that if you could not understand what it was about there was no point in explaining it to you. In general I agree with this, music, the greatest of all abstract arts makes its own impact and requires familiarity rather than explanation for its understanding and I think the same goes not only for abstract painting but for all visual art, a picture means what it appears to mean to the viewer, whatever the artist had in mind, a picture is its own message. This means that any interpretation of a picture, however ill informed, is a valid one, and particularly so in the case of Francis Bacon who declines to give any explanation of his art, and so I am emboldened to step in where angels fear to tread.

To spend one's life in medicine is to be engaged in a constant attempt to understand the processes involved in human health and disease. It is difficult to avoid drawing parallels in other fields, and one instinctively goes about solving a problem case in a clinical way—first of all get a good history.

Francis Bacon gave an interview to Shusha Guppy (1) reported in the Daily Telegraph in 1984 when he was 75. John Russell (2) has written a book about him. He was born in Dublin in 1909, his father was an Army Officer who later became a horse trainer. At the outbreak of War in 1914 he moved to London to work at the War Office, however the family never really settled in London and moved back and forth from Ireland. Francis Bacon suffered from asthma as a child and never had any proper schooling, an attempt to settle him at a boarding school at Cheltenham failed because he ran away. Though a gambler and a drinker his father adopted a high moral tone and when at the age of 16 he found his son trying on his mother's underwear he banished him from home. From that time on he led a life of drifting, when he was 20 he went to live in Berlin, a city, according to Russell 'laid on its back', later he drifted to Paris, and then back to London. He told Shusha Guppy "I didn't do anything much, I had one or two small jobs, but generally I drifted. I confess I was not at all honest, I would rent rooms and then disappear when I had to pay the rent, and I would live on theft and other people's kindnesses. What you call morality has grown on me with age." He admits his homosexuality and says "In many ways it is an affliction and limiting, but there it is. I am not at all unhappy about it and never have been. My father was a descendant of the Elizabethan Francis Bacon and the family have always called one son Francis. But funnily enough the original Francis Bacon was said to be a homosexual too—in *Aubrey's Brief Lives*—but Aubrey was a gossip." "I didn't really paint until I was over 30". He was rejected for National Service during the War because of his asthma. His early years were precarious, in poverty, with a background of gentility but without formal education, rejected by his family, depending on the help of friends, unable to form a stable marital relationship. He must have felt an outcast from society and much misery must have been his lot.

In 1944 he painted *Three Studies for Figures at the Base of a Crucifixion*. These were exhibited at the Lefevre Gallery along with works by Henry Moore, Mathew Smith and Graham Sutherland. They were creatures, part animal, part human that seemed to express evil, suffering and degradation. They compelled attention and from this time on he has continued to produce haunted visions depicting terror, mutilation, corruption and claustrophobia. The Art World is at his feet. He is acclaimed as the first British painter since Turner and Constable to achieve universal recognition. He is a cult figure in France, his last exhibition in Paris attracted 8,000 people in the first 24 hours and the police had to cordon off a whole street.

John Russell's well illustrated book provides the opportunity for a 'careful physical examination' and leafing through its pages one begins to discern a possible explanation for the symptomatology. It was Freud of course who introduced the concept of the Subconscious Mind which is the repository of forgotten memories and from which basic instinctual drives emerge. Glimpses into the subconscious mind occur in dreams, Bacon is said to spend much time in daydreaming, and his pictures have the quality of horrific dreams. Perhaps he descends into the abyss of his subconscious mind and emerges with his strange visions.

Bacon has given some hints as to his method of working. "What one requires is intelligence and awareness just to the edge, and beyond that to trust to instinct." "Real painting is a mysterious and continuous struggle with chance". "I think that painting today is pure intuition and luck and taking advantage of what happens when you splash the bits down". Two early pictures *Figure Study 1 and 2* show bodies lying under overcoats, one under an umbrella, recalling homeless nights in the open. A strange series of screaming Popes, variations of Velasquez's *Portrait of Pope Innocent X* could represent unwelcome homilies from the clergy attempting to bring him from the error of his ways. The picture *Lying figure with hypodermic syringe 1963* suggests that drug induced hallucinations may be a factor in his strange visions. Six of the illustrations in Russell's book show male figures, sometimes grotesquely distorted, lying prone one on top of the other leaving little room for doubt that a more literal rendering could be regarded as pornography. This is obviously a favourite theme. Two pictures illustrated in the book *Three figures in a room 1964* and *Triptych 1973* show naked male figures crouching upon lavatory seats, suggesting perhaps that a bowel evacuation may be a preliminary or a sequel to the acts depicted in the pictures in the previous group. In the triptych mentioned above another male figure appears to be heaving over a wash basin, this is also the theme of *Figure at a wash basin 1976*—no doubt a toxic postlude to some kind of orgy. Many of the illustrations show bodies or portions of bodies, mutilated, corrupted, liquefied, usually lying on mattresses under bare electric light bulbs, recalling doss houses. There are many named portraits in which the subject though grossly mutilated is probably recognisable to the 'next of kin'. Nearly all the subjects are confined by bare walls in a distorted perspective and sometimes they are surrounded by a kind of cage. These pictures conjure up appalling visions. In painting these horrors Bacon has perhaps achieved a Freudian psychoanalysis; he has emerged from his own abyss, but even yet the terrors are not exorcised. In old age he is honoured and admired by the cognoscenti. Yet he is indifferent to recognition or possessions, or even to the fate of his work. Rothenstein quotes him as saying "How can I take an interest in my work when I don't like it?" He also states that the only interest Bacon takes in his completed works is destructive: hundreds of his canvasses have been slashed and burnt. I feel his visions are autobiographical and personal but they also comment on the human condition. John Russell offering his own philosophical comment says "What if the world is one huge killing pound in which we pass the time while waiting for our turn in the arena? What if the act of love is a mimed death scene in which one partner steals the life of another?" In a sense his pictures are pathological specimens, they are also cries of despair.

Whether or not these speculations on the sources of Bacon's art have any validity, they do not answer the second question—why is his art not only accepted but hailed as the work of genius? In no civilisation and at no period, as far as I am aware, have representations of homosexual intercourse been acceptable or regarded as anything but pornography. Mutiliations of the human body, when not descriptive of events, whether real of fictitious, and purely 'for art's sake' are of course commonplace since Picasso, but Bacon's agonised corruptions, torsions, defacements, putrefactions of the body seem to be uniquely horrible.

John Russell, in his book already quoted says 'Our final impression of a major work by Bacon is that of the rehabilitation of beauty', he speaks of 'controlled grandeur', he observes that Bacon 'reinvented the human body' and also speaks of 'jewelled slime'. Such comments leave one speechless, do words have any meaning? Incidentally although Bacon's homosexuality is dealt with in Shusha Guppy's article in the Sunday Telegraph, Russell in his 'authoritative' and well illustrated book makes no mention of it. John Russell is not just an admirer of Bacon's art, he is a pundit in the art world, formerly art critic of the Sunday Times he is now art critic of the New York Times. He is a High Priest in the Temple of Art, preaching the gospel to the faithful. Am I the only surviving reactionary? My initial reaction was to regard Bacon as a highbrow horror comic and to shrug him off, but this will not do. There is nothing comic about Bacon, his work is simply and profoundly horrible.

I cannot avoid drawing another pathological parallel, society is losing some important immune defence reactions and the acceptance of Bacon's art is a sign of the acceptance of much else that is malignant, in fact we may be acquiring an immune deficiency syndrome. However, I think another pathological analogy may be nearer the mark. Bacon seems to have no imitators, the artists, if not the pundits, have recoiled, antibodies have been produced. If the exhibitors of blank canvases, coiled ropes and building bricks represent the *reductio ad absurdum* of Art, Bacon represents the *reductio ad nauseam*, a low point from which any further movement must be upwards. Nevertheless, Bacon's pictures now form part of the permanent collections of the world's art galleries and will be an enduring testimony to the taste of our times.

**BIBLIOGRAPHY**

1. GUPPY, SHUSHA, (1984) Sunday Telegraph Magazine, No. 419.
2. RUSSELL, JOHN, (1979) 'Francis Bacon.' Oxford University Press, World of Art Series.
3. ROTHENSTEIN, JOHN, (1962) Rothenstein, John. Introduction to Catalogue, Francis Bacon Exhibition, Tate Gallery.

